# Using an age-at-onset phenotype with interval censoring to compare methods of segregation and linkage analysis in a candidate region for elevated systolic blood pressure

**DOI:** 10.1186/1471-2156-4-S1-S84

**Published:** 2003-12-31

**Authors:** Karen A Kopciuk, Laurent Briollais, Florence Demenais, Shelley B Bull

**Affiliations:** 1 Samuel Lunenfeld Research Institute of Mount Sinai Hospital, 60 Murray Street, 5th Floor, Toronto, Ontario, M5G 1X5, Canada; 2 Department of Public Health Sciences, Faculty of Medicine, University of Toronto, Toronto, Ontario, Canada; 3 INSERM EMI00-06, Tour EVRY 2, 523, Place des Terrasses de l'Agora, 91034, France

## Abstract

**Background:**

Genetic studies of complex disorders such as hypertension often utilize families selected for this outcome, usually with information obtained at a single time point. Since age-at-onset for diagnosed hypertension can vary substantially between individuals, a phenotype based on long-term follow up in unselected families can yield valuable insights into this disorder for the general population.

**Methods:**

Genetic analyses were conducted using 2884 individuals from the largest 330 families of the Framingham Heart Study. A longitudinal phenotype was constructed using the age at an examination when systolic blood pressure (SBP) first exceeds 139 mm Hg. An interval for age-at-onset was created, since the exact time of onset was unknown. Time-fixed (sex, study cohort) and time-varying (body mass index, daily cigarette and alcohol consumption) explanatory variables were included.

**Results:**

Segregation analysis for a major gene effect demonstrated that the major gene effect parameter was sensitive to the choice for age-at-onset. Linkage analyses for age-at-onset were conducted using 1537 individuals in 52 families. Evidence for putative genes identified on chromosome 17 in a previous linkage study using a quantitative SBP phenotype for these data was not confirmed.

**Conclusions:**

Interval censoring for age-at-onset should not be ignored. Further research is needed to explain the inconsistent segregation results between the different age-at-onset models (regressive threshold and proportional hazards) as well as the inconsistent linkage results between the longitudinal phenotypes (age-at-onset and quantitative).

## Background

Hypertension is a common yet complex disorder. Genetic and environmental factors interacting over time are thought to be important in its development. Many segregation and linkage studies utilize data from families collected at a single time point. These cross-sectional studies will therefore include individuals who will become hypertensive in the future, but who now have blood pressure (BP) measurements considered to be within a normal range.

To address the longitudinal aspect of this disorder, Levy et al. [1] conducted a genome-wide scan to locate chromosomal regions linked to high BP, using the largest 332 Framingham Heart Study families. We note several points related to their methods. First, the construction of a longitudinal SBP phenotype for the 8478 subjects used the mean SBP measurement, based on a minimum length of follow-up and subject to age restrictions, with adjustment for BMI. The residuals from a model that regressed the within-subject mean SBP on the corresponding difference of the mean age and body mass index (BMI) for each subject from the sample means formed the longitudinal SBP. Second, observations from subjects who were being treated for high blood pressure were included in the study. Their observed BP measurements were adjusted using a nonparametric transformation to yield values expected to reflect their untreated BP measurements. Third, identity-by-descent (IBD) sharing for untyped individuals with phenotype information was inferred using SOLAR software [2]. Lastly, tests for linkage were conducted using variance components model as implemented in SOLAR.

The purpose of this current study is threefold: to assess the impact of interval censoring; to compare outcomes from two methods for segregation analyses using a longitudinal phenotype for elevated SBP based on age-at-onset; and to evaluate the evidence for linkage of this new phenotype to two markers found by Levy et al. Three age-at-onset phenotypes were constructed to address the interval-censored nature of the data, since ignoring this data feature can lead to inaccurate conclusions in standard survival analyses [3].

## Methods

### Study subjects

The Framingham Heart Study has been described in detail previously [4,5]. The salient feature of the study that we attempt to address is the periodic nature of the examinations for the study subjects. Individuals enrolled when the study began in 1948 had examinations repeated every 2 years. For subjects in the second cohort, which included the offspring of the first cohort and their spouses, the second examination took place 8 years after their enrollment. Subsequent examinations occurred every 4 years, with a final examination taking place 24 years after the first one. Since families were initially enrolled without regard for their hypertension status, they represent a random population-based sample and no correction for ascertainment was used.

### Age-at-onset systolic BP phenotypes

The outcome of interest in this investigation is the age at which systolic blood pressure (SBP) first exceeds 139 mm Hg or when treatment for hypertension begins. Because individuals treated for hypertension were classified as having high SBP, imputation was not necessary. However, since treatment could be initiated or SBP could have become elevated at any time between the previous visit where the SBP was found to be below the threshold and the current visit where it was found to be above, an age interval was created over which this event could have occurred. Individuals experiencing elevated SBP during the follow-up period could have three different ages at onset: the upper end-point is the age when elevated SBP or treatment was recorded, the lower end-point was the age at the previous visit when blood pressure was measured and the midpoint was the average of the two end-point ages.

In addition to interval censoring, the age-at-onset of high SBP was also subject to right censoring when SBP was always found to be less than 140 mm Hg during follow-up. The sole age recorded for censored observations was the earliest of age at death or end-of-study. Measured covariates included the fixed baseline covariates of sex and cohort (original or offspring), while body mass index (BMI), and daily alcohol and cigarette consumption were treated as time-dependent covariates.

The number of individuals in the data set formed by combining both cohorts with the age-at-onset information was 2884. About 50% of these individuals (*n *= 1444) experienced elevated SBP during the follow-up period. The average upper age-at-onset for this group was 50.8 years (S.D. = 11.9 years), with onset ranging from 13 to 97 years. These descriptive values were very similar for the lower age-at-onset (average = 49.6 years, S.D. = 11.8 years, range 12–97 years) and, by construction, for the age midway between these extremes (average = 50.2 years, S.D. = 11.8 years, range 13–97 years).

### Segregation analyses

We applied two methods of segregation analyses: a proportional hazards (PH) model with a frailty term [6,7] and a regressive threshold model developed to account for age-at-onset data [8,9].

We used three different definitions for age for onset (upper, midpoint, lower), and either time-fixed covariates (sex, cohort) alone or in conjunction with time varying covariates (BMI, daily alcohol and cigarette consumption). The baseline hazard function was approximated by a step function over six age intervals (<40, 40–45, 45–50, 50–55, 55–60, >60). Using available software, maximum likelihood estimation (MLE) of model parameters was carried out assuming Hardy-Weinberg equilibrium, mendelian transmission probabilities, and either a dominant or recessive unmeasured gene segregating within families.

#### Proportional hazards model

This model, which is implemented in GAP, uses a frailty term for an unmeasured diallelic major gene (MG) [6,7]. The baseline hazard function is approximated by a step function on a user-specified number of intervals and all covariates (measured and unmeasured) are assumed to act multiplicatively on this baseline. Under Hardy-Weinberg equilibrium assumption, the population distribution of genotypes depends only on the (unknown) allele frequency. The transmission probabilities for each genotype are fixed under the mendelian inheritance assumption.

#### Regressive threshold model (RTM)

The regressive model [10] is constructed by specifying a regression relationship between each person's phenotype and a set of explanatory variables including genotype at a MG, phenotype of antecedents to account for unspecified sources of residual family dependences (due to other genes and/or shared environmental factors), and observed covariates. For binary traits, Demenais [8] proposed an alternative formulation of the regressive logistic models that assumed an underlying liability to the disease with a threshold determined from the morbid risk in the population. The RTM has been recently extended to analyze diseases with variable age-of-onset by introducing time-dependent thresholds [9]. The variation of risk with age is modelled assuming a piece-wise constant hazard function. Models can accommodate time-dependent covariates and include different parametric functions to express the variation of the hazard with time. In this study, we assume a constant displacement *t *and dominance *d *for each age-class *k *(*k *= 1,...,6). The RTM formulation was implemented in the package REGRESS [11,12], which incorporates the regressive approach in the ILINK program of the LINKAGE package [13]. Standard errors for model parameters are available in this package but it was not possible to invert a large matrix of second partial derivatives. Bootstrap samples can provide these variability estimates, but require substantial running time.

### Linkage analyses

These analyses were limited to the 52 largest families (more than 20 individuals) due to the substantial computation time required to fit the RTM. About 30% of these 1537 individuals had genotype and phenotype information. Generally, about 40% with no missing phenotype or genotype information experienced high SBP during the follow-up period individuals (versus 50% in the data set with 330 families). However, the age discrepancies (average, S.D., and range) between the data with complete phenotype information and this subset appear to be minimal.

Due to sparse data, rare alleles were grouped for markers that had more than five alleles in our analyses. Although the REGRESS software can conduct multipoint linkage analyses for a small number of markers, we conducted two-point analyses to reduce computation time. Parameter estimates from the segregation analyses assuming a recessive trait were fixed in the linkage analyses.

## Results and Discussion

### Segregation analyses

Direct comparison of estimates obtained from the two methods is not appropriate, however, their sensitivity to the choice of age-at-onset is. Since there were small differences between the log likelihoods for the dominant and recessive models, and the recessive models were used in the subsequent linkage analyses because they had the largest log likelihoods, only the results for the recessive mode of inheritance are reported (Table 1). More extensive model-fitting to determine a best-fitting model for these data was not carried out.

In the PH models, the allele frequency estimates become progressively smaller as the age-at-onset changes from the lowest possible value to the highest for each person, but the differences are small relative to the standard errors. In the RTM models, the major gene parameter estimates tend to become progressively larger as the age-at-onset increases.

The estimates for the measured covariates generally seem robust to the choice of age-at-onset, except that the RTM parameter estimates for the sex and cohort variables are much smaller for the lower age-at-onset model. The parameter estimates are much smaller when the lowest age-at-onset is used in both the two-covariate and five-covariate models than in the comparable middle and upper ages-at-onset models. Based on likelihood ratio statistics, the five-variable models were clearly an improvement over the two-variable models.

**Figure 1:**
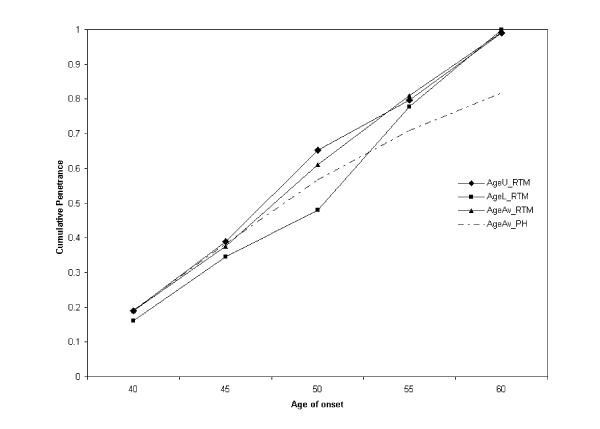
Estimation of the cumulative penetrance among carriers for recessive mode of inheritance models

The estimated baseline penetrance risk functions (corresponding to male carriers in Cohort 1) for the recessive RTM are very similar for all three ages at onset, except at age 50 (Figure 1). The PH curve for average age was similar to that for RTM up to age 50.

### Linkage analyses

The LOD scores for chromosome 17 marker GATA25A04 from the recessive RTM are generally quite small (0.03, 0.02, 0.11 for upper, midpoint, lower) compared to the 3.8 reported by Levy et al. The corresponding recombination fractions (0.18, 0.50, 0.50) apparently also depended on the age-at-onset definition, however it is difficult to draw conclusions about the effect of interval censoring when there is no linkage signal. Findings for chromosome 17 marker ATC6A06 were similar. None of the significant findings by Levy et al. [1] were replicated in our study. In addition to the differences in the construction of the longitudinal phenotypes and the linkage methods used, other possible explanations for these discrepant findings include the numbers of families included in the analyses and the handling of missing BP measurements.

## Conclusions

These segregation and linkage analyses suggest that interval censoring for the age-at-onset should not be ignored, the choice of segregation method can affect some results, and the type of longitudinal phenotype or linkage method can affect linkage detection. Using different ages at onset affected parameter estimates in the segregation analyses related to the unknown major gene, as well as the estimates of cumulative penetrances, and the estimated recombination fraction in the linkage analyses. The segregation results using lower age-at-onset appeared to be most discordant with the other two age-at-onset values, but further research is needed to provide practical recommendations.

**Figure 2:**
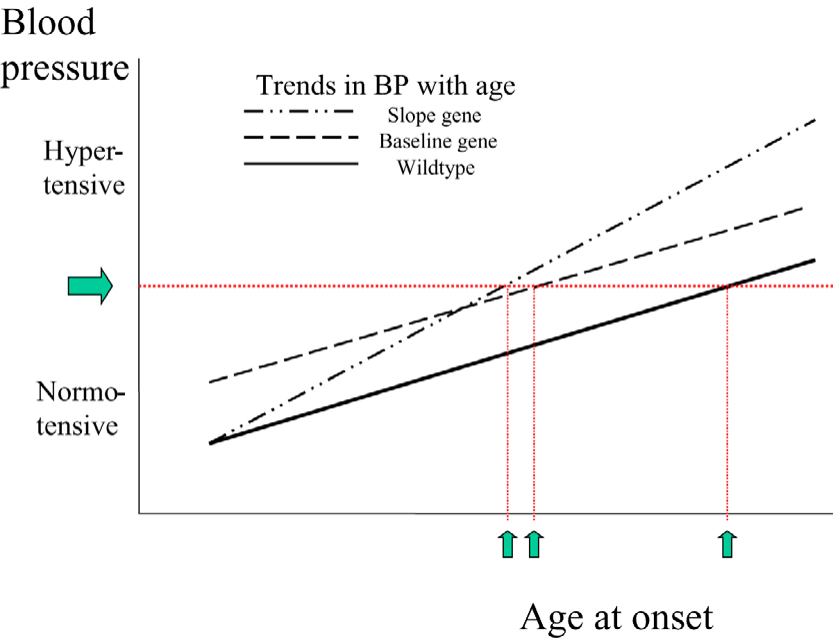
Relationship between age-at-onset and average BP measurements models

Further research is needed to understand the relevant differences between the two segregation methods and the two longitudinal phenotypes employed in this study. We believe both constructed phenotypes should be picking up similar longitudinal aspects of the onset of disease. In the Levy et al. paper, each individual's average SBP would be below or above the threshold of 139 mm Hg (indicated by large green arrow on y-axis in Figure 2) which, in the age-at-onset model, would correspond to a censored or observed event, respectively (assuming a sufficient length of follow-up for the age-at-onset phenotype; ages-at-onset indicated by smaller green arrows on x-axis in Figure 2). The curves in the plot represent three different lifetime measures of SBP: 1) a person with a wild-type gene, 2) a person with a baseline gene who has elevated SBP early in life relative to person with a wildtype gene and maintains this difference through-out his/her lifetime, and 3) a person with "slope" gene who initially has the same SBP as a person with a wild-type gene, but whose SBP increases at a higher rate over his/her lifetime. By modeling time to event more directly, we supposed that an age-at-onset approach would also detect strong linkage signals due to either early gene effects or effects that emerge with age.

Model-free methods may be more appropriate than model-based methods for genome scans of complex diseases. However, the latter may be of interest for confirming linkage results from model-free methods. It may also be better in some cases to estimate jointly all the model parameters, rather than in two stages. Simulation studies directly comparing longitudinal phenotypes and various methods of analyses (model-based versus model-free) would help disentangle these differences and would guide selection of phenotypes and analysis methods for other outcomes with variable age-at-onset. Although the event of elevated SBP is of clinical and therapeutic interest, and its definition serves to circumvent the problem of BP levels being modified by treatment, it is likely that information from the quantitative measure is lost, yielding less sensitive tests for genetic effects and larger sample size requirements for comparable power. For disease phenotypes in which quantitative measures are limited or unreliable, further development of methods and software for time-to-event outcomes and related longitudinal phenotypes would be welcome.

## Figures and Tables

**Table 1:** Segregation Analysis Results for Three Age-at-onset Traits. Recessive Trait Parameter Estimates (S.E.) from Proportional Hazards Models(GAP) and Regressive Threshold Models (REGRESS)

	Parameter					
	
	Two Covariates			Five Covariates		
	
Model	Upper^A^	Mid-point^B^	Lower^C^	Upper^A^	Mid-point^B^	Lower^C^
Proportional Hazards						
Allele Frequency	0.76(.05)	0.78(.05)	0.81(.05)			
Major Gene^D^	1.40(.13)	1.41(.14)	1.37(.15)			
Sex^E^	0.23(.07)	0.23(.06)	0.22(.06)			
Cohort^F^	0.52(.07)	0.52(.06)	0.53(.06)			
-logL	6728	6764	6867			
Regressive Threshold						
Allele Frequency	0.50	0.55	0.52	0.55	0.51	0.51
Major Gene^D^	0.96	0.83	0.60	1.52	1.32	1.74
Sex^E^	0.29	0.29	0.21	0.11	0.10	0.01
Cohort^F^	0.01	0.01	-0.08	-0.02	0.01	-0.08
BMI^G^				0.05	0.05	0.05
Smoking^H^				0.02	0.02	0.05
Drinking^I^				0.01	0.01	0.01
Parent-offspring corr	0.15	0.13	0.16	0.16	0.11	0.12
Sib-Sib corr	0.00	0.00	0.16	0.08	0.06	0.10
-logL	6558	6679	6600	6477	6604	6537

^A^Upper value from age interval. ^B^Value at midpoint of interval. ^C^Lower value from age interval. ^D^Major gene effect is measured by the displacement (see Methods). ^E ^Sex variable (female vs. male). ^F^Cohort variable (offspring vs. original). ^G^Body mass index (weight/height^2 ^in kg/m^2^). ^H^Daily cigarette consumption. ^I^Daily alcohol consumption (g/day).
